# Pathological Complete Response in Locally Advanced *ALK* Fusion Gene–Positive Lung Adenocarcinoma following Salvage Surgery: A Case Report and Literature Review

**DOI:** 10.70352/scrj.cr.25-0238

**Published:** 2025-09-26

**Authors:** Kazuki Ohta, Naoki Haratake, Shinkichi Takamori, Yuta Abe, Eika Kudo, Takahiro Sato, Kosuke Kamada, Daiki Noda, Miyuki Abe, Yohei Takumi, Michiyo Miyawaki, Tsutomu Daa, Kenji Sugio, Atsushi Osoegawa

**Affiliations:** 1 Department of Thoracic and Breast Surgery, Oita University Faculty of Medicine, Yufu, Oita, Japan; 2 Department of Medical Oncology, Dana-Farber Cancer Institute Harvard Medical School, Boston, MA, USA; 3 Department of Thoracic Surgery, Oita Prefectural Hospital, Oita, Oita, Japan; 4 Department of Diagnostic Pathology, Oita University Faculty of Medicine, Yufu, Oita, Japan

**Keywords:** locally advanced non–small cell lung cancer (NSCLC), anaplastic lymphoma kinase (ALK), *ALK* fusion gene–positive NSCLC, alectinib, ALK tyrosine kinase inhibitor (ALK-TKI), salvage surgery, pathological complete response (pCR)

## Abstract

**INTRODUCTION:**

Alectinib, a 2nd-generation anaplastic lymphoma kinase–tyrosine kinase inhibitor (ALK-TKI), is an established 1st-line therapy for advanced *ALK* fusion gene–positive non–small cell lung cancer (NSCLC). However, the role of salvage surgery following alectinib for locally advanced disease remains uncertain.

**CASE PRESENTATION:**

A 41-year-old woman was diagnosed in the postpartum period with Stage IIIA (cT1cN2M0) *ALK* fusion gene–positive lung adenocarcinoma. She received 1st-line alectinib, achieving a 55.3% reduction in tumor size over 11 months. Subsequent salvage surgery revealed a pathological complete response with no residual tumor cells. During postoperative follow-up off alectinib, recurrence was observed 20 months after surgery, with new brain and pulmonary metastases. Reintroduction of alectinib achieved renewed disease control, and the patient has remained progression-free for 23 months since restarting therapy.

**CONCLUSIONS:**

This case highlights the potential role of salvage surgery following alectinib in locally advanced *ALK* fusion gene–positive NSCLC. Furthermore, it suggests that maintenance ALK-TKI therapy after salvage surgery might be associated with a reduced risk of recurrence. Further studies are warranted to optimize perioperative ALK-targeted strategies.

## Abbreviations

A^3^
: the segment 3 arterial branch
ALK
: anaplastic lymphoma kinase
CI
: confidence interval
CR
: complete response
EGFR
: epidermal growth factor receptor
^18^F-FDG
: fluorine-18-fluorodeoxyglucose
FISH
: fluorescence *in situ* hybridization
IASLC
: International Association for the Study of Lung Cancer
MPR
: major pathological response
NSCLC
: non–small cell lung cancer
pCR
: pathological complete response
PD
: progressive disease
PFS
: progression-free survival
PR
: partial response
RECIST v 1.1
: Response Evaluation Criteria in Solid Tumors version 1.1
SD
: stable disease
TKI
: tyrosine kinase inhibitor
UICC
: Union for International Cancer Control

## INTRODUCTION

*ALK* fusion gene–positive lung cancer accounts for approximately 2%–7% of NSCLC cases, predominantly affecting younger, nonsmoking patients.^1–3)^ The phase III J-ALEX trial and ALEX trial established alectinib, a 2nd-generation ALK-TKI, as the standard 1st-line therapy for advanced *ALK*-rearranged NSCLC, demonstrating significantly improved PFS (~34 versus ~10 months with crizotinib) and a lower incidence of central nervous system progression compared to crizotinib.^4,5)^ While the efficacy of alectinib is well documented in unresectable Stage IIIB/IV *ALK*-rearranged NSCLC and as adjuvant therapy in resected Stage IB–IIIA *ALK*-rearranged NSCLC,^4–6)^ the role of salvage surgery following alectinib for potentially resectable, locally advanced *ALK* fusion–positive NSCLC remains under investigation. We present a case of Stage IIIA *ALK* fusion gene–positive lung adenocarcinoma treated with alectinib followed by salvage surgery resulting in a pCR. Despite this favorable response, the recurrence observed after postoperative discontinuation of alectinib supports the hypothesis that maintenance ALK-TKI therapy after salvage surgery may be beneficial in reducing the risk of recurrence.

## CASE PRESENTATION

**Fig. 1:**
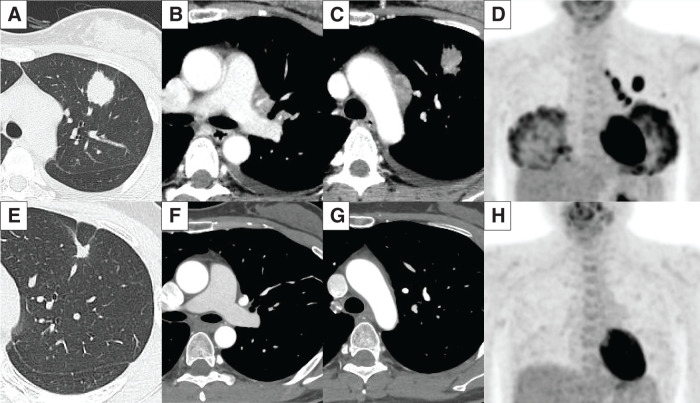
(**A–D**) Pretreatment clinical stage was Stage IIIA (cT1cN2M0) according to the 8th edition of UICC/IASLC TNM classification. (**A**) Contrast-enhanced chest CT scan showed a 25-mm irregular nodule in the left upper lobe segment 3. (**B**) Enlarged left hilar lymph nodes at stations 12u and 13 were present, suggesting possible invasion of A^3^ and the left pulmonary artery. (**C**) Enlarged mediastinal nodes at stations 5 and 6. (**D**) Fluorine-18-fluorodeoxyglucose PET-CT showed intense metabolic activity in both the primary lesion (the maximum standardized uptake value rising from 11.3 to 15.0) and the involved lymph nodes, with no evidence of distant metastasis. (**E–H**) After 11 months (342 days) of treatment, corresponding to restaging as Stage IA2 (ycT1bN0M0). (**E**) The primary tumor had shrunk by 55.3%—from 25 to 11 mm. (**F**, **G**) The previously enlarged nodes regressed. (**H**) There was complete resolution of fluorine-18-fluorodeoxyglucose uptake. A^3^, the segment 3 arterial branch; IASLC, International Association for the Study of Lung Cancer; UICC, Union for International Cancer Control

A 41-year-old woman (nonsmoker) in late pregnancy was incidentally found to have a pulmonary nodule in the left upper lobe during preoperative assessment for an elective Caesarean section. Her medical history included chronic kidney disease and an ovarian cyst. There was no family history of malignancy. Contrast-enhanced chest CT scan showed a 25-mm irregular nodule in the left upper lobe segment 3 (**Fig. 1A**). Enlarged left hilar lymph nodes at stations 12u and 13 were present, suggesting possible invasion of the A^3^ and the left pulmonary artery (**Fig. 1B**). Enlarged mediastinal nodes were also noted at stations 5 and 6 (**Fig. 1C**). ^18^F-FDG PET-CT showed intense metabolic activity in both the primary lesion (the maximum standardized uptake value rising from 11.3 to 15.0) and the involved lymph nodes, with no evidence of distant metastasis (**Fig. 1D**). Contrast-enhanced brain MRI confirmed the absence of intracranial metastases. Based on these findings, the clinical stage was determined to be Stage IIIA (cT1cN2M0) according to the 8th edition of UICC/IASLC TNM classification.

**Fig. 2:**
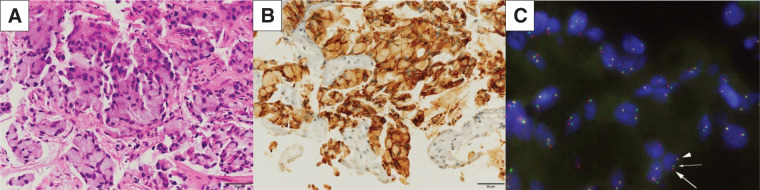
(**A**) A transbronchial lung biopsy revealed adenocarcinoma growing in a nested pattern, with signet-ring cells containing abundant intracytoplasmic mucin (hematoxylin and eosin staining, high-power magnification). (**B**) Immunohistochemistry (Ventana ALK D5F3 assay) showed strong granular cytoplasmic ALK positivity (high-power magnification). (**C**) Fluorescence *in situ* hybridization with an ALK break-apart probe (Vysis; Abbott, Abbott Park, IL, USA) confirmed an *ALK* gene rearrangement in 88% of tumor cells. ALK, anaplastic lymphoma kinase

A transbronchial lung biopsy revealed adenocarcinoma growing in a nested pattern, with signet-ring cells containing abundant intracytoplasmic mucin (**Fig. 2A**). Immunohistochemical staining with a monoclonal antibody (D5F3; Ventana-Roche Diagnostics, Mannheim, Germany) revealed strong granular cytoplasmic ALK positivity (**Fig. 2B**). FISH with an ALK break-apart probe (Vysis; Abbott, Abbott Park, IL, USA) confirmed an *ALK* gene rearrangement in 88% of tumor cells (**Fig. 2C**). The tumor was also positive for programmed death-ligand 1, with a tumor proportion score of 40% (Dako 22C3 antibody).

Following delivery, alectinib was initiated as 1st-line therapy at 600 mg twice daily. A PR, as defined by RECIST v 1.1, was achieved within 1 month and maintained for 10 months. After 11 months (342 days) of therapy, the primary tumor had shrunk by 55.3%—from 25 to 11 mm (**Fig. 1E**). The previously enlarged nodes regressed, and there was complete resolution of ^18^F-FDG uptake, corresponding to restaging as Stage IA2 (ycT1bN0M0) (**Fig. 1F**–**1H**). An R0 resection was achieved via video-assisted thoracoscopic left upper lobectomy with systematic nodal dissection, performed 5 days after the final dose of alectinib.

**Fig. 3:**
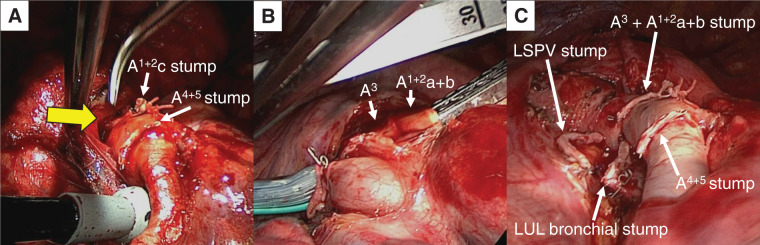
(**A–C**) Intraoperative view: Fibrous adhesions were observed between the hilar lymph nodes and the A^3^ segmental artery, but no extranodal invasion was apparent. (**A**) These nodes were dissected together with the adjacent vascular sheath (yellow arrow: A^3^). (**B**) Following the removal of stations 12u and 13, A^3^ and A^1+2^a+b were divided using a surgical stapler. (**C**) Completion of left upper lobectomy with systematic nodal dissection. A^3^, the segment 3 arterial branch; LSPV, left superior pulmonary vein; LUL, left upper lobe

**Fig. 4:**
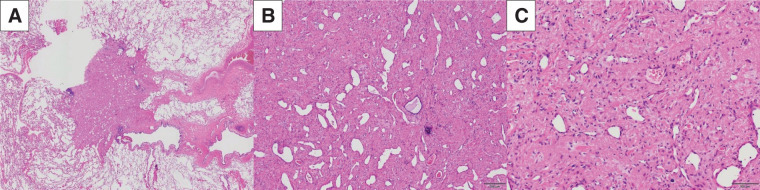
(**A**) Pathological examination showed a 7 × 5-mm eosinophilic nodular lesion within the lung (HE staining, low-power magnification). (**B**, **C**) Histopathological examination of the resected specimen revealed a pathological complete response, with no viable tumor cells in the lung parenchyma or lymph nodes. The lesion displayed prominent fibrous proliferation and collapsed alveolar structures (**B**: HE, low-power magnification; **C**: HE, high-power magnification). HE, hematoxylin and eosin staining

At surgery, marked tumor regression induced by alectinib was evident. Fibrous adhesions were observed between the hilar lymph nodes and the A^3^ segmental artery, but no extranodal invasion was apparent. These nodes were dissected together with the adjacent vascular sheath, permitting routine division of A^3^ (**Fig. 3A**–**3C**). Histopathological examination of the resected specimen revealed pCR, with no viable tumor cells in the lung parenchyma or lymph nodes (**Fig. 4A**–**4C**). The patient was discharged on POD 8 without complications. Alectinib was discontinued after surgery due to financial considerations.

**Fig. 5:**
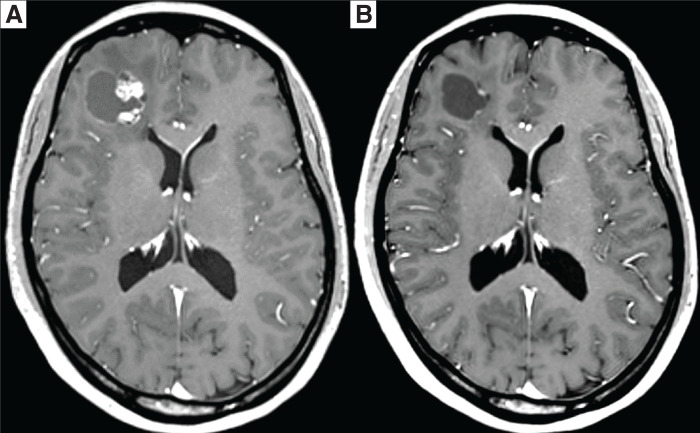
(**A**) Enhanced brain MRI (T1-weighted imaging) revealed an abnormal signal area with peripheral enhancement in the right frontal lobe, consistent with recurrent brain metastasis. (**B**) One month after reinitiation of alectinib, the metastatic lesion shrank markedly, and contrast enhancement was reduced on follow-up MRI.

The patient remained disease-free for 20 months after surgery, after which surveillance imaging detected new metastases in the brain and pulmonary regions (**Fig. 5A**). Reintroduction of alectinib achieved rapid disease control, and she has since remained progression-free for 23 months to date (**Fig. 5B**).

## DISCUSSION

We report a case of initially unresectable Stage IIIA *ALK*-rearranged lung adenocarcinoma that was discovered incidentally during evaluation for a Caesarean section. Alectinib induced a profound tumor response, enabling salvage surgical resection with a pCR. However, discontinuation of alectinib after surgery was followed 20 months later by metastatic recurrence in the brain and lung. Reinitiation of alectinib rapidly re-established disease control, and the patient has remained progression-free for 23 months since restarting therapy. This case highlights the potential role of salvage surgery following alectinib in locally advanced *ALK* fusion gene–positive NSCLC and suggests a possible benefit to continuing ALK-targeted therapy postoperatively.

Salvage surgery is increasingly considered for patients with residual or recurrent disease after definitive nonsurgical therapy (e.g., chemotherapy and/or radiotherapy), but data on its feasibility and outcomes following ALK-TKI therapy remain scarce. We identified 31 published cases of salvage surgery performed after alectinib therapy (**Table 1**).^7–23)^ The median patient age was 58 years (range, 29–72 years) (**Table 2**). Of the 31 patients, 18 (58%) were male and 11 (35%) were female (sex was unreported in 2 cases). With respect to smoking history, 11 patients (35%) were never-smokers and 7 (23%) were former smokers (smoking status was not reported in 13 patients). Most patients presented with Stage III disease (clinical stage: IIB in 6%, IIIA in 39%, IIIB in 45%, IVA in 6%, and IVB in 3%), and none were Stage I–IIA. The median duration of preoperative alectinib was 109 days (range, 30–540 days), with 68% of patients receiving ≥9 weeks of therapy. Radiological responses to alectinib were as follows: CR in 3 patients (10%), PR in 20 (65%), SD in 2 (6%), and PD in 2 (6%) (response not reported for 4 cases). Notably, among 28 patients with N2/N3 nodal involvement, 19 (68%) were downstaged to N0 and underwent R0 resection (**Table 1**).

**Table 1:** Literature review of case reports on salvage surgery following alectinib

Age/sex	Smoking status	cTNM (the 8th edition)	Radiological response	Duration of preoperative therapy	Surgery (approach)	ypTNM	pCR	Postoperative complications	Adjuvant therapy	Status	Postoperative follow-up	Recurrence site (time)
40/F, our case	Non	T1cN2M0 (IIIA)	PR (55.3%)	343 days	Left upper lobectomy (thoracotomy)	T0N0M0	Yes	None	None	Alive (recurrence)	43 mo	Pulmonary and brain (20 mo)
65/F^7)^	N/A	TXN2M0 (IIIA)	N/A (N/A)	90 days (3 mo)	Left lower lobectomy + lingular segmentectomy (N/A)	N/A	No (MPR)	None	None (refused)	Alive	5 mo	N/A
71/M^8)^	N/A	T3N2M0 (IIIB)	PR (95%)	308 days	Lobectomy (thoracotomy)	T0N0M0	Yes	None	None	Alive (recurrence)	16 mo	Brain (9 mo)
65/N/A^9)^	Former (20 py)	T3N2M0 (IIIB)	N/A (N/A)	84 days (12 w)	Right middle lobectomy (N/A)	N/A	No (MPR)	None	None	Alive (recurrence)	12 mo	Multiple lymph nodes (12 mo)
46/M^10)^	Non	T3N2M0 (IIIB)	PR (47%)	56 days	Left lower lobectomy (N/A)	T1aN0M0	No (MPR)	None	N/A	Alive	N/A	N/A
64/M^11)^	Non	T3N2M0 (IIIB)	PR (N/A)	44 days	Right lower lobectomy (VATS)	N/A	Yes	None	Ceritinib	Alive	N/A	N/A
66/M^11)^	Former (10 py)	T4N2M0 (IIIB)	PR (N/A)	109 days	Right upper lobectomy (VATS)	N/A	No (MPR)	None	Alectinib	Alive	N/A	N/A
67/M^12)^	Non	T4N2M0 (IIIB)	CR (100%)	84 days (150 mg BID)	Left upper lobectomy (VATS)	T1aN0M0	No	N/A	N/A	N/A	N/A	N/A
47/F^13)^	Current (1.25 py)	T2bN3M0 (IIIB)	PD (N/A)	180 days (6 mo)	Left upper lobectomy (N/A)	T3N0M0	No	None	Lorlatinib	Alive	22 mo	None
41/M^8)^	N/A	T1cN2M0 (IIIA)	PR (75%)	415 days	Lobectomy (RATS)	T1a(m)N0M0	No (MPR)	Anemia	Lorlatinib	Alive	17 mo	None
34/F^14)^	N/A	T3N2M0 (IIIB)	CR (100%)	182 days	Lobectomy (thoracotomy)	TXN0M0	Yes	N/A	Alectinib (+ perioperative chemo)	Alive	77 mo	None
66/M^8)^	N/A	T3N2M0 (IIIB)	PR (95%)	214 days	Lobectomy (thoracotomy)	T0N0M0	Yes	Prolonged air leak (Grade I)	Alectinib	Alive	13 mo	None
72/M^8)^	N/A	T4N2M0 (IIIB)	PR (90%)	194 days	Lobectomy (thoracotomy)	T0N0M0	Yes	Chylothorax (Grade II)	Alectinib	Alive	11 mo	None
56/M^8)^	N/A	T4N2M0 (IIIB)	PR (95%)	42 days	Lobectomy (VATS)	T0N0M0	Yes	None	Alectinib	Alive	4 mo	None
49/F^8)^	N/A	T2N2M0 (IIIA)	SD (25%)	254 days	Pneumonectomy (thoracotomy)	T3N2M0	No	None	Alectinib	Alive	2 mo	None
60/M^8)^	N/A	T3N2M0 (IIIB)	CR (100%)	178 days	Bilobectomy (VATS)	T0N0M0	Yes	None	Alectinib	Alive	5 mo	None
55/F^8)^	N/A	T3N2M1b (ADR) (IVA)	PR (90%)	292 days	Lobectomy (VATS)	T2N0M0	No (MPR)	None	Alectinib	Alive	5 mo	None
64/M^8)^	N/A	T2bN2M1a (PUL) (IVA)	PR (47.6%)	170 days	Lobectomy (thoracotomy)	T1bN2M0	No (MPR)	None	Alectinib	Alive	4 mo	None
62/M^8)^	N/A	T2aN2M0 (IIIA)	PR (35%)	56 days	Lobectomy (thoracotomy)	T0N2M0	No (MPR)	None	Alectinib	Alive	31 mo	None
62/M^15)^	Former (N/A)	T2aN2M0 (IIIA)	PR (N/A)	56 days	Left upper lobectomy (VATS)	T0N2M0	No (MPR)	None	Alectinib	Alive	N/A	N/A
61/N/A^9)^	Former (5 py)	T1bN2M0 (IIIA)	N/A (N/A)	42 days (6 w)	Left upper lobectomy (RATS)	T0N0M0	Yes	None	Alectinib	Alive	3 mo	None
59/M^16)^	N/A	T3N2M1c (BRA, HEP, OTH) (IVB)	PD (N/A)	540 days (18 mo)	Left upper lobectomy (N/A)	N/A	No	N/A	Alectinib	Alive (with disease)	N/A	Liver metastases get enlarged without alectinib therapy
41/M^17)^	Non	T2aN3M0 (IIIB)	PR (69%)	420 days (14 mo)	Left upper lobectomy (VATS)	N/A	Yes	None	Alectinib	Alive	29 mo	None
52/F^18)^	Non	cT4N2M0 (IIIB)	PR (86%)	75 days (2.5 mo)	Left pneumonectomy (VATS → Thoracotomy)	N/A	Yes	N/A	Alectinib	Alive	12 mo	None
51/M^19)^	Non	T2aN2M0 (IIIA)	PR (42.2%)	45 days	Right upper lobectomy (VATS)	N/A	No	None	Alectinib	Alive	5 mo	None
58/F^20)^	Non	T2bN2M0 (IIIA)	PR (90%)	56 days	Right middle and lower bilobectomy (VATS)	T0N0M0	Yes	None	Alectinib	Alive	8 mo	None
29/F^21)^	Never	T2aN2M0 (IIIA)	N/A (N/A)	60 days (2 mo)	Right lower lobectomy (VATS)	T2aN0M0	No (MPR)	None	Alectinib	Alive	1 mo	None
56/F^22)^	Non	T2aN2M0 (IIIA)	PR (75%)	91 days (13 w)	Right upper lobectomy (thoracotomy)	T0N0M0	Yes	None	Alectinib	Alive	18 mo	None
51/M^23)^	Former (heavy)	T3N0M0 (IIB)	PR (66%)	210 days (30 w)	Left lower lobectomy (VATS)	T0N0M0	Yes	None	Alectinib	Alive	13 mo	None
48/M^23)^	Non	T3N0M0 (IIB)	PR (70%)	224 days (32w)	Right middle lobectomy (VATS)	T0N0M0	Yes	None	Alectinib	Alive	12 mo	None
62/F^21)^	Former (N/A)	T4N0M0 (IIIA)	SD (26%)	30 days (1 mo)	Right upper lobectomy + wedge resection (VATS)	T4N0M0	No	None	Adjuvant chemo only	Alive	22 mo	None

The staging is according to the 8th edition of UICC/IASLC TNM classification.

BID, twice a day; CR, complete response; cTNM, clinical TNM classification; F, female; IASLC, International Association for the Study of Lung Cancer; M, male; mo, months; MPR, major pathological response; N/A, not available; pCR, pathological complete response; PD, progressive disease; PR, partial response; py, pack-years; RATS, robot-assisted thoracoscopic surgery; SD, stable disease; UICC, Union for International Cancer Control; VATS, video-assisted thoracoscopic surgery; w, weeks; ypTNM, yield pathological TNM classification

**Table 2:** Summary of the literature review

Factors	N = 31
Age (years), median (range)	58 (29–72)
Sex, no. (%)	
Male	18 (58)
Female	11 (35)
N/A	2 (6)
Smoking status, no. (%)	
Never	11 (35)
Former/Current	7 (23)
N/A	13 (42)
Initial disease stage, no. (%)	
I–IIA	0 (0)
IIB	2 (6)
IIIA	12 (39)
IIIB	14 (45)
IVA	2 (6)
IVB	1 (3)
TNM classification, regional lymph nodes, no. (%)	
N0	3 (10)
N1	0 (0)
N2	24 (77)
N3	2 (6)
N2 or N3 (IIIB, not reported)	2 (6)
Duration of preoperative alectinib therapy (days), median (range)	109 (30–540)
Preoperative alectinib interruption (days), median (range)	6 (0–32) ^†^
Adverse events, no. (%)	
None	11 (35)
Grade 1–2	7 (23)
Grade 3	1 (3)
Grade 4–5	0 (0)
N/A	12 (39)
Radiological response (RECIST v 1.1), no. (%)	
Complete response	3 (10)
Partial response	20 (65)
Stable disease	2 (6)
Progressive disease	2 (6)
N/A	4 (13)
Type of resection, no. (%)	
Lobectomy	27 (87)
Bilobectomy	2 (6)
Pneumonectomy	2 (6)
Surgical approach, no. (%)	
Thoracotomy (including 1 conversion)	10 (32)
VATS	14 (45)
RATS	2 (6)
N/A	5 (16)
Pathological response, no. (%)	
pCR (0% viable tumor)	15 (48)
MPR (≤10% viable tumor)	25 (81)
Others	6 (19)
Adjuvant therapy, no. (%)	
None	4 (13)
Alectinib	21 (68)
Lolratinib	2 (6)
Ceritinib	1 (3)
Chemotherapy alone	1 (3)
N/A	2 (6)

^†^Data were not reported for 11 cases. The staging is according to the 8th edition of UICC/IASLC TNM classification.

IASLC, International Association for the Study of Lung Cancer; MPR, major pathological response; N/A, not available; pCR, pathological complete response; RATS, robot-assisted thoracoscopic surgery; RECIST v 1.1, Response Evaluation Criteria in Solid Tumors version 1.1; UICC, Union for International Cancer Control; VATS, video-assisted thoracoscopic surgery

Postoperative management varied. Twenty-one patients continued alectinib after surgery (including 1 who initiated alectinib after perioperative chemotherapy).^14)^ Two patients received lorlatinib (1 of whom discontinued after 8 months due to adverse events).^13)^ One patient received ceritinib, and another received cisplatin–pemetrexed chemotherapy. Four patients received no maintenance therapy, and in 2 cases, the postoperative treatment was not reported (**Table 2**). The median follow-up among reported cases was 12 months (range, 1–77 months).

Preoperative alectinib was generally well tolerated. No treatment-related adverse events were reported in 11 patients (35%), and only Grade 1–2 toxicities occurred in 7 patients (23%), consisting chiefly of gastrointestinal upset,^10,20)^ transient aminotransferase elevations,^9,23)^ and hemolytic anemia^9)^ (**Table 2**). Adverse event data were not reported for 12 patients. Severe adverse events (Grade 3–4) were uncommon; only 1 patient developed Grade 3 interstitial pneumonitis 44 days after starting alectinib, which resolved with a 6-day course of methylprednisolone, allowing surgery to proceed.^11)^ Dense fibrosis and adhesions at prior tumor sites and nodal involvements were frequently described. In 1 report, fibrous adhesions from an initially invasive hilar tumor prevented separation of the lobe bronchus from pulmonary veins, necessitating conversion from video-assisted thoracoscopic left lower lobectomy to an open pneumonectomy 75 days after preoperative alectinib.^18)^ In another, after 45 days of alectinib, dense scar tissue around a paratracheal nodal station precluded safe dissection due to excessive surgical risk.^19)^ No additional severe intraoperative difficulties were reported. These findings suggest that dense fibrous adhesions may persist at the sites of the primary tumor or lymph node involvement, even after radiologically confirmed downstaging. No perioperative deaths or severe postoperative complications were reported, supporting the feasibility of salvage surgery in well-selected patients despite these technical challenges.

Pathological responses to preoperative alectinib appear favorable compared to historical results with chemotherapy. Among the 31 cases reviewed, MPR (≤10% viable tumor) and pCR (no viable tumor) were achieved in 25 (81%) and 15 (48%) patients, respectively (**Table 2**). This pCR rate exceeds those reported for neoadjuvant chemotherapy in NSCLC (approximately 4%–15%).^24,25)^ By comparison, in the ALNEO phase II trial (GOIRC-01-2020-ML42316), all 33 enrolled patients completed neoadjuvant alectinib, and 28 (85%) proceeded to surgery.^26)^ MPR was observed in 15 patients (46%; 90% CI, 31%–61%), and pCR in 4 patients (12%; 95% CI, 3%–28%). Objective radiographic response was documented in 22 patients (67%). The higher pathological response rates in our series likely reflect selection bias (only patients with favorable responses underwent surgery) as well as longer preoperative treatment durations, given that ALNEO limited alectinib to 8 weeks.

The optimal duration of preoperative ALK-TKI therapy remains undefined. In advanced NSCLC, responses to ALK-TKI are typically seen within ~2 months.^27)^ The NAUTIKA1 and ALNEO trials showed that 8 weeks of neoadjuvant alectinib achieved both efficacy and tolerability,^26,28)^ although severe adverse events such as Grade 3 interstitial pneumonitis have been reported. In our 31-case review, treatment duration ranged from 1 to 18 months, with no clear association between length of alectinib therapy and surgical difficulty or postoperative complications (see **Table 1**). Consistent with prior reports, prolonged preoperative alectinib therapy did not invariably make resection more difficult; in cases where technical challenges arose, these were attributable to the extent of initial tumor invasion rather than treatment duration. In the present case, surgery was not performed at the time the patient first achieved a PR. However, considering the risks of prolonged therapy—including potential serious adverse events, development of drug resistance, and possible loss of surgical eligibility—it may be prudent to proceed with surgery once a sufficient response (e.g., a PR) is achieved and warrants further prospective evaluation.

Similarly, neoadjuvant trials of EGFR-TKIs in resectable NSCLC have typically employed 6–9 weeks of preoperative therapy, with MPR rates of 11%–25% and pCR rates of 0%–9%.^29–32)^ These studies provide a valuable benchmark for comparison. A 16-week course of almonertinib (3rd-generation EGFR inhibitor) did not show a marked increase in pathological response rates.^33)^ This suggests that simply lengthening targeted therapy beyond ~2 months may not yield additional benefit.^34)^ Cross-trial comparisons should be interpreted with caution because of differences in study designs, patient populations, response assessments, and treatment durations.

A key question is whether to continue ALK-TKI therapy after salvage surgery. A recent retrospective analysis of patients undergoing salvage resection (including 3 *ALK*-rearranged and 33 *EGFR*-mutated cases) suggested a potential benefit of salvage surgery following TKI therapy; however, the 3-year recurrence-free survival after surgery was only 22% (95% CI, 9%–40%).^35)^ In our series, among the 4 patients (including the present case) who achieved MPR or pCR but did not resume an ALK inhibitor after surgery, 3 developed distant metastases within 9–20 months of resection. By contrast, recurrences were uncommon among patients who received postoperative ALK-TKI maintenance (alectinib, ceritinib, or lorlatinib) during the available follow-up period. These observations suggest that—even when a pCR is achieved after salvage resection following preoperative alectinib—initially unresectable *ALK*-rearranged NSCLC remains prone to early distant relapse, highlighting the potential need for maintenance ALK-TKI therapy to reduce recurrence risk. This pattern aligns with results of the ALINA trial, which demonstrated that adjuvant alectinib significantly improved disease-free survival versus platinum-based chemotherapy in resected Stage IB–IIIA *ALK*-positive NSCLC (hazard ratio, 0.24; 95% CI, 0.13%–0.45%; *p* = 0.001).^6)^ Although ALINA evaluated planned adjuvant therapy, its findings support the strategy of continuing ALK inhibition after salvage surgery. Taken together, a fixed 2-year course of maintenance alectinib following salvage surgery may prolong PFS even in patients who achieve pCR; however, this inference should be interpreted cautiously given the retrospective, observational nature of the data.

In the present case, financial constraints were associated with differences in therapeutic decision-making and with less favorable clinical outcomes, highlighting the potential importance of enhanced support programs to reduce cost-related barriers to care.

## CONCLUSIONS

In summary, alectinib for locally advanced *ALK*-rearranged NSCLC produced a profound tumor regression that enabled R0 salvage resection with a pCR. Early distant relapses occurred when alectinib was not continued postoperatively, whereas reinitiation of alectinib achieved durable disease control. A synthesis of reported cases indicates that preoperative alectinib is associated with high pathological response rates and frequent nodal downstaging, without adding undue surgical risk apart from fibrosis-related challenges. These observations support the feasibility and potential benefit of performing surgery after a response to ALK-TKI therapy, and suggest that postoperative maintenance ALK-TKI might be associated with a reduced risk of recurrence even after pCR. Prospective studies are needed to define the optimal duration of preoperative therapy, the timing of surgery, and the role of maintenance ALK-TKI therapy after surgery to further improve outcomes for locally advanced *ALK* fusion gene–positive NSCLC patients.

## DECLARATIONS

### Availability of data and materials

The datasets supporting the conclusions of this article are available from the corresponding author upon reasonable request.
